# Diversity Awareness in Medical Education: An Innovative Training with Visual Reflection Tools

**DOI:** 10.5334/pme.1080

**Published:** 2023-11-02

**Authors:** Winny Ang, Liesbeth Verpooten, Benedicte De Winter, Katrien Bombeke

**Affiliations:** 1 Child and adolescent psychiatrist, communication skills teacher and PhD student in the Department of Family Medicine and Population Health at the University of Antwerp, Wilrijk, Belgium; 2 General practitioner and communication skills teacher in the Department of Family Medicine and Population Health at the University of Antwerp, Wilrijk, Belgium; 3 Head of the skills lab, vice-dean faculty Medicine and Health Sciences, University of Antwerp, Wilrijk, Belgium; 4 General practitioner and professor communication skills in the Department of Family Medicine and Population Health at the University of Antwerp, Wilrijk, Belgium

## Abstract

Dealing with a diverse population is one of the most challenging topics in medicine, with consequences for health disparities as evidenced by poorer health of marginalised groups. An urgent need exists to build a diversity-responsive curriculum in medical education. At the core of such a curriculum are experiential learning and a focus on self-awareness and reflexivity via small group trainings. This Show and Tell paper describes the development and qualitative evaluation of such a training, that was implemented at the University of Antwerp in Belgium, presenting answers to some of the gaps and challenges described in the literature. This training is guided by three visual reflection tools – the kaleidoscope, the iceberg and the communication compass – to inspire learners on how to deal with the diversity of their future patients. The content, method, and educational aim of this hands-on training are described. We discuss some of the challenges the educational methods pose on reflexivity and awareness, looking at the lessons learned based on participants’ feedback. While the visual reflection tools offer a dynamic space to broaden the way we look at patients, it remains imperative to create a safe environment for discussing tensions, sharing difficult topics and being aware of different voices. Taking time (space for discussion, small groups, training of faculty) and allowing for continuous reflection of the educators are key in the development of diversity-responsive education.

## Background & Need for Innovation

In health care, dealing with a diverse population is one of the most challenging topics of this era with persistent disparities leading to poor health and early mortality in marginalised groups [1,2,3]. Physicians lack knowledge of minority patients [4]. Furthermore, they have limited awareness of differences in communication styles, doctor-patient relationship expectations and the diverse meanings of health and illness leaving them unprepared to address these issues [5].

In healthcare professions education (HPE), there is great variability in the diversity education offered to trainees as well as uncertainty about ‘what works’. For example, a recent comparative case study of cultural competence training at 15 medical schools in the United States [6] did not find a clear definition or pattern of cultural competence training as a one-size-fits-all approach to curricular components and style of presentation. The findings indicate that longitudinal integration, tone of content delivery and experiential learning are potentially important. Indeed, the process towards becoming a diversity-responsive physician is complex, involving an orientation that recognises dignity and autonomy of patients plus a focus of providing high quality of care to a pluriform society across culture, gender and class [7].

To train diversity responsive physicians, we must move beyond the acquisition of cultural competence, knowledge, and expertise towards developing self-awareness, practical communication skills, and interpersonal teamwork across differences [8]. Muntinga and colleagues present four overarching learning objectives toward this goal: knowledge, skills, patient-physician communication and reflexivity [9]. Future clinicians must learn to use their own identity, both in terms of self-understanding and with an awareness of how they appear to others given this social historical background, as a tool to explore patients’ identity, the meaning of illness, the social context of illness and adaptation, and the clinical relationship itself. This self-awareness is the focus of experiential learning and clinical training [10]. Although the relevance of experiential learning and awareness in a diversity-responsive education is clear [5,10], little is known about the concrete methods used to create and stimulate awareness in medical students’ learning processes. This paper describes the training sessions we developed at our medical school for an awareness-oriented diversity training.

## Goals of Innovation

Before 2011, diversity education at the University of Antwerp in Belgium consisted of a longitudinal, theoretical curriculum ‘Doctor & Society’ spanning the first five years of medical school. In early 2011, this was supplemented with one experiential ‘intercultural communication’ skills training, using migrant simulated patients (SP) and rather stereotyping roleplay scripts. Based on the literature [11] and our own educational struggles [12], we felt the need to develop a learning method for learners to focus more on awareness as essential in the diversity-responsive curriculum. Main educational goals were for students to obtain a broader view on ‘diversity’ and raise awareness of personal premises and assumptions.

## Steps taken for Development and Implementation of Innovation

### Context

**Figure 1:**
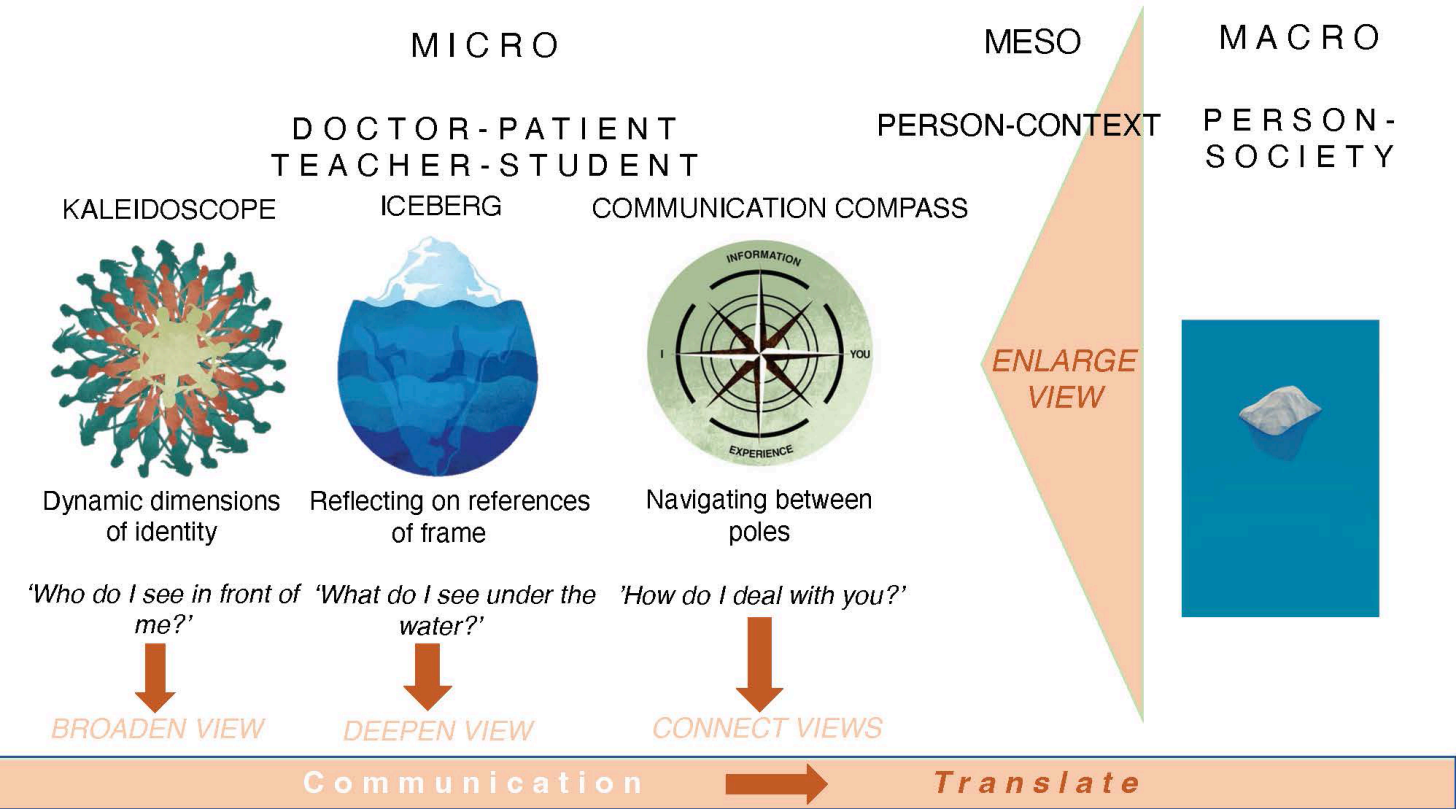
Three visual reflection tools utilized for teaching diversity responsiveness.

At the University of Antwerp, the six-year medical curriculum includes communication skills training at a ratio of 6 trainings of 3 hours per year. Furthermore, there is formal evaluation (OSCE) and informal evaluation (tutorships). In 2013, we moved the ‘intercultural’ session from Year 4, where it was surrounded by ‘challenging communication’ sessions (e.g., breaking bad news), to Year 1, where it is now preceded by basic relationship-building sessions (e.g., active listening). It is important to create awareness from the first year on, as a basis to build on other communication skills in subsequent years. For this training, which we renamed into a ‘diversity’ session, we developed three visual reflection tools (Figure 1). In 2015, we added an extra skills training with UNsimulated patients (UP) instead of simulated patients (SP), also in Year 1. UP are trained in giving feedback as SP, but do not ‘play a role’. They are invited to talk about their personal dimensions of identity and the three visual reflection tools are integrated [12].

### Set-up of the present training (2 sessions)

The first session has a *learning goal* of raising awareness of one’s own assumptions and gaining insight into a diversity-responsive social history-taking. *The educational method* is guided by three visual reflection tools (Figure 1) to inspire students on how to reflect on dealing with diversity in patients. We explain the meaning and use of these tools below. *Practical characteristics:* a 3-hour session with maximum 20 participants and one/two trained facilitators.

The second session is a role play with UP, with as a *learning goal* skills-practice for diversity-responsive active listening and asking sensitive, explorative questions about the different dimensions of identity. It is the first step of social history-taking in a consultation. *The educational method* is based on ALOBA (agenda-led, outcome-based analysis) feedback [13]. In this learner-centred method, the personal learning agenda directs the feedback discussion by encouraging self-assessment and descriptive feedback based on observation. It is key to present a supportive and safe environment. The visual tools are used during this session to stimulate more awareness concerning unconscious bias, beliefs and stereotypes in a concrete and hands-on way. *Practical characteristics*: a 3-hour small group session with six students, three rotating UP, and one facilitator. *Assessment:* diversity-responsive active listening skills and asking explorative questions are assessed in a summative OSCE station with written feedback.

In both sessions, the facilitators are part of our communication skills teaching team. In addition to being trained in ALOBA, they were all trained to use the visual reflection tools.

#### Visual reflection tool 1: Kaleidoscope

##### Content

The kaleidoscope is an instrument containing loose bits of coloured material between two transparent plates and two mirrors placed so that changes of position of the material are reflected in an endless variety of patterns. We use it as a metaphor for the different dimensions of identity. With each turn we do not reset it to a former or final state, but a new one that reflects the here-and-now positions of the pieces we must work with. For example, in certain situations sexual identity is more on the foreground for someone, yet sometimes it is irrelevant.

The experiential exercise is Caleidoscopia [14], an existing card game based on a person’s many identity dimensions (e.g., phase of life, ethnicity, social class, …).

##### Method of the exercise

Participants are grouped in teams of 4–5 persons and given a set of cards with the eight dimensions of identity. They receive the following instructions:

1. Everyone takes a card and introduces themselves based on the dimension on the card.
2. The other group members ask questions sensitively to find out more about this dimension of the person’s identity.

After the exercise there is space to share experiences. The facilitator links the experiences to a mini-lecture about identity and dealing with different dimensions of identity. The concept of the kaleidoscope can spotlight diversity and similarities, raising awareness in seeing the multidimensionality rather than the monodimensionality.

##### Educational aim

Learning to ask explorative questions is a substantial element of getting acquainted with the diversity in yourself and others. The kaleidoscope becomes a tool to ‘broaden’ the view of yourself and others. This exercise underlies the following skills training with the UP and introduces how to take a patient’s social history.

#### Visual reflection tool 2: Iceberg

##### Content

Weaver [15] explains the layers of culture through the image of an iceberg. The surface culture is the part of the iceberg that is above water (10%) and is visible to others and easily identifiable (language, behaviour, customs, traditions). At this level of culture, others are primarily aware of cultural differences. However, there is a deeper level of culture (90%) below the surface that is often difficult to view or identify (beliefs, values).

Culture is like an iceberg, because so much goes undetected. The influence of culture on the elements of communication needs to be explicitly explored rather than taken for granted or ignored. More on a microlevel, the iceberg is the metaphor of every person’s frame of reference, which in turn reveals individuals’ worldview perspective.

##### Method of the exercise

A thought-provoking story, presented in Table 1, is used to evoke small group discussions. The story is standardized such that every character makes certain choices based on personal values and beliefs.

**Table 1:** A thought-provoking story illustrating the concept of the ‘iceberg’ as part of the ‘diversity’ session [16].


**The king, the queen, and her lover** The king loves the queen but has to go on a long journey. He suspects the queen has a lover, and orders the castle guard to kill her if she leaves the castle during his absence. He tells his wife about this before departing from the castle. When the king leaves, the queen’s lover contacts her and urges her to come to him. She says she cannot come, since the guards have been ordered to kill her if she leaves. Her lover says that he desperately needs to see her, and that it’s important that she come the following night. The queen talks to one of the guards, who says he will protect her and help her get out of the castle and back inside, provided she returns by midnight. The queen meets her lover and comes back to the castle on time. The guard who promised to help her has fallen asleep. Another guard sees her as she tries to enter the castle, and kills her. Order the characters of the story, based on how responsible they are for the queen’s death: – The king – The queen – The lover – The guard who falls asleep – The guard who kills her


After the exercise the facilitator creates space to share experiences and links it to a mini-lecture about the iceberg and the different frames of reference we all have. The visible part is clear but one’s beliefs and values are situated underwater, invisible and often unconscious, until friction occurs. The concept of the iceberg can raise awareness of your own and the other’s frame of reference, and the challenges of bridging different frames of reference; this is part of every unique patient-caregiver encounter.

The concept of the iceberg is a baseline to talk about the biases everyone has about/towards others, and stimulates trying to know more about the other person’s unseen side. This iceberg is also useful towards explaining concepts of illness and disease, taking into account your own biomedical frame of reference and your patient’s perspectives.

##### Educational aim

Using the concept of the iceberg to discover more about the sometimes-hidden part of yourself (what’s my own perspective?) and the other (what’s happening underwater?) is the most important focus. The iceberg becomes a tool to ‘deepen’ the view you have of yourself and the other.

#### Visual Reflection Tool 3: Communication Compass

##### Content

The concept of the communication compass was developed by Maex [17] to support the processes of mutual understanding between patient and doctor. It provides a handhold to analyse specific situations and to look at possibilities and pitfalls. There are two axes: the axis of perspectives (patient-doctor, or more generally: you-me) and that of information versus experience.

The first point in communication is to recognise the perspective of the other (which you can never access directly but only indirectly by communication) without losing sight of your own perspective [15]. The other axis is about navigating between information (objective, e.g., facts) and experience (subjective, e.g., feelings/perception). In communication, both aspects are present simultaneously. It is important to differentiate these two elements and give space to both information and experience. For example, the question of a palliative patient ‘doctor, how long do I have?’ can be both a request for information (days, months?) or merely an expression of feelings (e.g., anxiety).

##### Method

A clinical case from the participants can be used to demonstrate how the compass works. For example: As a student you consider gender equality important. During an internship you notice a female patient who doesn’t say a lot during the consultation, her husband is always talking; even when the doctor tries to get the woman involved, he answers. You are irritated by the situation. This convinces you of the idea that their religion tends to facilitate gender inequalities.

Using the compass, these are possible elements to reflect on: What are the two perspectives? Does the clinician know what the perspective of the patient and the husband is? Does the patient know what the perspective of the clinician is? Looking at the second axis, questions arise such as: The notion that religion facilitates gender inequalities, is that information or is that perception?

##### Educational aim

The compass is a dynamic tool that creates space for different perspectives of the doctor and the patient and examines how the views can be more ‘connected’, searching for a shared narrative where both information and experience have their space and mutually interact.

We provide an overview of the three visual reflection tools in Table 2.

**Table 2:** Overview of the three visual reflection tools and learning goals.


	CONTENT		LEARNING GOALS		
	
	EXPERIENTIAL EXERCISE	MINI-LECTURE	KNOWLEDGE	SKILLS	AWARENESS

KALEIDOSCOPE	Caleidoscopia: card game exploring different dimensions of identity in small groups	Kaleidoscope: dynamic dimensions of identity	– Dynamic perspective on identities – Multidimensionality of identities – Diversity in every person (yourself and the other) – Intersectionality	– The art of asking questions, listening, and talking – Reflecting on your own diversity by looking at the different dimensions of identity	– Curiosity – Taboos – Bias – Unexpected turns

ICEBERG	Provoking story: discussion in small groups, exploring conscious/unconscious values, norms, etc.	– Iceberg: reflecting on frames of reference	– Concept of having a frame of reference – Ontological loupe: is there one single truth? – Insight into how unconscious elements (under the waterline) determine and direct your behaviour – Impact of context on the iceberg: how the environment influences the way someone expresses their conscious/unconscious values, norms, etc.	– Reflecting on your own frame of reference = reflexivity – Dealing with different opinions in a group – Exploring other opinions leads to understanding that you yourself and others have conscious/unconscious behaviour determinants – Exploring different perspectives in a group	– Curiosity – Bias – Different frames of reference lead to impact at the emotional level – Unexpected turns

COMMUNICATION COMPASS	Case study from participants: discussion in small groups. Looking at the challenges of the case study by exploring the different perspectives	Communication compass: navigating between poles and searching for shared narratives	– Compass helps achieve a mutual understanding and thus bridge different perspectives/frames of reference – Effective communication includes both Information and Experience	– Navigating between poles/different positions of the compass: dealing with diversity – Reflecting on/dealing with your boundaries and those of the other person – Bridging between different perspectives and persons	– Curiosity – Unique person-to-person interaction – Dynamics in every interaction – Boundaries (self & other)


## Evaluation of Innovation

Since the first training in 2011, we have continuously evaluated the training based on PDCA-circles (Plan, Do, Check, Act) [18]. We structurally collected feedback from students, SP/UP and the team of facilitators to adapt and improve our training. We also collected feedback from participants of the visual reflection session at international conferences from 2018–2022. Apart from informal feedback, in 2017 we started to collect data as part of a PhD project, exploring how medical students learn to deal with diversity, taking in account the society where they live in. We conducted semi-structured interviews and focus groups with 28 students and 7 UP between 2017 and 2020 after ethical approval (Antwerp University Hospital EC). The findings of this PhD study are beyond this paper’s scope, but specifically to evaluate our innovation, we selectively analysed the data on training and tools to explore respondents’ reaction to the training (Kirkpatrick level 1) and from their perspectives, what they learned (Kirkpatrick level 2) [19]. Further research is needed to examine higher level outcomes, such as behavior change and patient impact.

## Critical reflections on our process

These evaluations helped us critically examine our educational process. The most important reflections are described below, illustrated by participant quotes.

### The danger of stereotyping and looking at the ‘exotic’ other

“Are we so different that there has to be a separate training with us?” (SP, 2011)

In our own history of creating a diversity-responsive curriculum, the earliest sessions ‘Intercultural Communication’ were skills-trainings embedded in ‘challenging communication’ sessions. That addressed rather stereotypical knowledge focusing on ‘ethnicity’ and skills-training with SP that all had a migration background, the so-called ‘exotic other’ [20]. This stimulated us to conceptualise a broad concept of diversity, taking in account other dimensions of identity (e.g. gender, sexual orientation, class). It remains a challenge to find a balance in underfocusing/overfocusing on ethnicity and race and still be aware of the risks of both. The kaleidoscope is a useful tool to pay attention to the different dimensions of identity. The uniqueness of the person based on the recognition that all people are ‘equal and different at the same time’ emerges as a dialectic between universality and particularism [21] leading to patient-centeredness.

### The importance of using the three visual reflection tools

“You can be very stuck on one path being straightforward. But you can also try to understand the other. There will always be differences and you can never be on the same page the whole time. The compass can help navigate this process.” (Student, Year 1, 2020)

As we mentioned before, we felt the need to broaden the way we look at the patients and how we obtain information without stigma. The visual reflection tools offer this dynamic space to students and teachers. Integrating visual imagery and metaphorical thinking in this reflective teaching methodology enables teachers to have a clear objective of the learning goals and know how to proceed and attain them through the process of teaching and learning. This stimulates the thought process of the learner and helps retain the information, prioritising and rearranging ideas to create meaningful concepts [22].

This reflection stimulated us to continue developing the tools using drawings of a professional illustrator and developing a 3D model of the compass.

### The challenges of the use of tools

“I’m not a fan of too many models. Instead of one metaphor, three in a row …. Eventually you feel like you have to study those three models more than actually see the point.” (Student, Year 1, 2017)

In the first sessions with the visual reflection tools, there was a risk to promote a model that emphasises mastery and one truth if you use the tools in a ‘rigid’ and ‘fixed’ way. This critical reflection induced more training for the facilitators to clearly understand and explain the meaning and challenges of the tools, to add enough demonstrative exercises, emphasizing the dynamic use of the tools and allow space for discussion.

### Creating a safer space for discussion

“I consider the exercises on awareness really valuable …. It stimulates you to think about values and norms, your own iceberg. There was space for discussion. There’s nothing you can get as much out of as a discussion.” (Student, Year 1, 2020)

Students perceive the training as a safe environment to share ‘difficult’ topics, allowing vulnerability. Reflecting on diversity can cause some tensions in persons themselves or between persons in the group. As a facilitator we had to learn to deal with these tensions and give space to other opinions and different voices. This stimulated us to be continuously aware that creating a safer space is key to navigate in/between these tensions.

### Learning from resistance

“Why do I have to adapt my attitude as a doctor? What are the boundaries between myself and the patient?” (Facilitator, 2015)

After a team debriefing of a session where students and facilitator had a tense discussion on a complex patient case, we decided to elaborate more on the communication compass as a tool. This stimulated us to give more time to reflect on the different perspectives of the healthcare provider and the patient, and likewise the facilitators and the learners, looking for a possible connection.

### The power of the experiential approach

“First, you get some theory but then you’re in front of a real patient and you realise: I have to ask personal questions. The impact of what you’ve learned will stay with you.” (Student, Year 1, 2017)

“What’s going to stick with me the most is the exercise with the king. Only then did I realise that everyone really thinks in different ways.” (Student, Year 4, 2017)

This reflection stimulated us to dedicate more teaching time to the experiential exercises instead of offering a merely knowledge or skills-based session.

### The necessity of taking time

“One session is too short to know the concepts, you can easily do a second session to practice with a simulated patient. Many people think they can swim, but they can’t. Awareness is important and takes time.” (Student, Year 4, 2017)

We do not believe in a one-shot diversity training and felt a 3-hour session introducing the tools seemed too short to facilitate awareness. This reflection stimulated us to organize two sessions: one experiential session to become familiar with the visual tools and a second 3-hour session to apply the tools by practicing through the interview with the UP. In the two sessions there is space for discussion. After this critical reflection, we also decided to longitudinally include the visual tools in other parts of the curriculum. Educators who consider implementing diversity-responsive education, need to take this investment of time into account (space for discussion, small groups, training of facilitators, faculty and UP). From a broader meso-perspective: taking time and a continuous reflection of the educators are important in the development of diversity-responsive education.
